# A Comprehensive Review of Magnesium Sulfate Infusion: Unveiling the Impact on Hemodynamic Stability During Laryngoscopy and Tracheal Intubation in Ear, Nose, and Throat Surgeries

**DOI:** 10.7759/cureus.57002

**Published:** 2024-03-26

**Authors:** Urvi Sawant, Jayashree Sen

**Affiliations:** 1 Anaesthesiology, Jawaharlal Nehru Medical College, Datta Meghe Institute of Higher Education & Research, Wardha, IND

**Keywords:** perioperative care, ent surgeries, tracheal intubation, laryngoscopy, hemodynamic stability, magnesium sulphate

## Abstract

This comprehensive review explores the potential of magnesium sulfate infusion in mitigating hemodynamic instability during laryngoscopy and tracheal intubation in ear, nose, and throat (ENT) surgeries. Hemodynamic fluctuations during these procedures pose challenges, and magnesium sulfate, with its vasodilatory, antiarrhythmic, and neuroprotective properties, emerges as a promising intervention. The review critically examines existing literature, emphasizing patient selection criteria, dosage protocols, and a comparative analysis with other hemodynamic stabilizers. Safety considerations, including known adverse effects and risk-benefit assessments, and monitoring and management strategies are elucidated. The implications for ENT surgery are discussed, highlighting the potential for enhanced hemodynamic management and individualized approaches. The review concludes with a call for continued research, emphasizing the ongoing evolution of understanding and practice incorporating magnesium sulfate into perioperative care. The insights offered aim to guide clinicians in navigating this dynamic landscape for improved patient outcomes in ENT surgeries.

## Introduction and background

Ear, nose, and throat (ENT) surgeries, encompassing ear, nose, and throat procedures, stand as integral components within the broader medical landscape. Maintaining hemodynamic stability during these surgeries is paramount as it significantly influences patient safety and overall surgical outcomes. Hemodynamic stability refers to the equilibrium within the cardiovascular system, ensuring optimal blood flow and organ perfusion [1]. The significance of maintaining hemodynamic stability in ENT surgeries cannot be overstated. These procedures often involve manipulations that can elicit significant physiological responses, particularly during laryngoscopy and tracheal intubation. Fluctuations in blood pressure, heart rate, and other hemodynamic parameters can pose considerable challenges, potentially leading to adverse events such as myocardial ischemia, arrhythmias, or cerebrovascular incidents [2].

Laryngoscopy and tracheal intubation, routine components of many ENT surgeries, present unique challenges. Introducing instruments into the airway and using anesthetic agents can trigger a cascade of physiological changes, including sympathetic stimulation and an increase in catecholamine release. Without careful management, these changes may result in hemodynamic instability, compromising patient safety and the success of the surgical intervention [3]. Various pharmacological interventions have been explored to mitigate the challenges posed by hemodynamic instability during ENT surgeries. One such intervention is the infusion of magnesium sulfate. Magnesium, a divalent cation, plays a multifaceted role in cellular physiology and has been investigated for its potential to modulate hemodynamic responses [4].

This comprehensive review aims to critically examine the impact of magnesium sulfate infusion on hemodynamic stability, specifically during laryngoscopy and tracheal intubation in ENT surgeries. By synthesizing existing literature, evaluating methodologies employed in previous studies, and analyzing key findings, this review aims to provide a nuanced understanding of the role of magnesium sulfate as a potential pharmacological agent in maintaining hemodynamic equilibrium during these critical moments in ENT procedures. The evidence synthesis will inform clinicians, researchers, and healthcare practitioners about the current state of knowledge, potential benefits, and areas for further exploration in this context.

## Review

Hemodynamic responses during laryngoscopy and tracheal intubation

Normal Physiological Changes

Laryngoscopy and tracheal intubation initiate phy and tracheal intubation [2ysiological responses as the airway is secured for anesthesia and surgery. Typically, these responses involve a rapid and transient increase in sympathetic activity, resulting in elevated heart rate, blood pressure, and systemic vascular resistance. These changes are part of the body's natural stress response to airway manipulation and the introduction of anesthetic agents [5]. Understanding the regular physiological changes is crucial for healthcare providers to differentiate between expected responses and those indicative of potential complications. While a certain degree of sympathetic activation is normal, excessive or prolonged responses can lead to hemodynamic instability, necessitating intervention [6].

Complications and Adverse Effects

Hypertension: Elevated blood pressure during medical procedures such as laryngoscopy and tracheal intubation can be attributed to sympathetic nervous system stimulation. This physiological response places additional strain on the cardiovascular system, leading to an increased workload for the heart and potential complications for patients with pre-existing hypertension or cardiovascular conditions. The heightened blood pressure may contribute to vascular damage and increase the risk of adverse events, emphasizing the need for effective strategies to manage and prevent hypertensive responses during these procedures [7].

Tachycardia and arrhythmias: The acceleration of heart rate, known as tachycardia, often accompanies the sympathetic stimulation triggered by laryngoscopy and tracheal intubation. When persistent and irregular, this increase in heart rate can lead to arrhythmias, disrupting the normal cardiac rhythm. Tachycardia and arrhythmias compromise cardiac function, affecting the heart's ability to pump blood efficiently. Such disturbances can have profound implications for patients, especially those with pre-existing heart conditions, highlighting the importance of interventions to mitigate these adverse cardiac effects during these critical medical procedures [8].

Hypotension: Paradoxical hypotension, characterized by a sudden and unexpected decrease in blood pressure, is a potential complication, particularly during the induction of anesthesia associated with laryngoscopy and tracheal intubation. This poses risks of inadequate perfusion to vital organs, potentially leading to organ dysfunction and compromising patient safety. Preventing and managing hypotension becomes crucial in ensuring optimal hemodynamic stability and minimizing the adverse consequences of inadequate blood flow to vital tissues [9].

Myocardial ischemia: The heightened cardiac workload resulting from the physiological responses to airway manipulation can create an imbalance between oxygen supply and demand, potentially leading to myocardial ischemia. In this scenario, the heart muscle may not receive sufficient oxygen, increasing the risk of tissue damage and cardiovascular events. Myocardial ischemia poses a severe threat, particularly in patients with pre-existing coronary artery disease, necessitating careful management strategies to maintain a balance between oxygen demand and supply during laryngoscopy and tracheal intubation [10].

Cerebrovascular incidents: Fluctuations in blood pressure, whether hypertensive or hypotensive, can profoundly affect cerebral perfusion, increasing the risk of adverse neurological events known as cerebrovascular incidents. Inadequate blood flow to the brain can result in conditions such as strokes or transient ischemic attacks. The impact on cerebral perfusion emphasizes the critical need for interventions that address cardiovascular stability and consider the potential consequences on neurological function, particularly in patients with a history of cerebrovascular diseases [11].

Importance of Maintaining Stability

Patient safety: Ensuring hemodynamic stability is paramount for patient safety during surgical procedures, particularly in laryngoscopy and tracheal intubation. Hemodynamic stability minimizes the risk of cardiovascular events, providing a foundation for the patient's overall well-being. This focus on safety is crucial, especially for individuals with pre-existing cardiovascular conditions, as it mitigates the potential for complications and enhances the overall safety profile of the surgical experience [12].

Optimal surgical conditions: Hemodynamic stability significantly creates an optimal surgical environment. Stable hemodynamics allow for a controlled setting, enabling surgeons to perform precise interventions with reduced interference from physiological fluctuations. This controlled environment is instrumental in facilitating surgical precision and minimizing the challenges associated with fluctuations in blood pressure and heart rate, ensuring the procedure's success and the patient's safety [13].

Postoperative outcomes: The impact of hemodynamic stability extends beyond the surgical phase to influence postoperative outcomes. Stability during the perioperative period is linked to favorable recovery trajectories, reduced postoperative complications, and enhanced patient comfort in the post-anesthesia care unit (PACU). By maintaining hemodynamic equilibrium throughout the surgical journey, healthcare providers contribute to positive postoperative experiences and the intervention's overall success [14].

Overall morbidity and mortality: Unstable hemodynamics during critical phases such as laryngoscopy and tracheal intubation have been associated with heightened morbidity and mortality rates. The destabilizing effects on the cardiovascular system can lead to adverse events, emphasizing the pivotal role of maintaining stability in improving overall patient outcomes. Addressing hemodynamic fluctuations becomes a central element in comprehensive patient care, impacting the immediate perioperative period and the broader spectrum of morbidity and mortality associated with surgical interventions [15].

Magnesium sulfate (MgSO_4_): properties and mechanisms

Chemical Composition and Characteristics

Magnesium sulfate, chemically represented as MgSO_4_, is a naturally occurring mineral with a range of physiological and pharmacological properties. It comprises magnesium ions (Mg²⁺) and sulfate ions (SO₄²⁻). The compound exhibits hygroscopic characteristics, readily absorbs water, and is commonly found in medical and non-medical applications [16]. MgSO_4_ in medicine spans various specialties, including obstetrics, neurology, and anesthesia, owing to its diverse effects on cellular function and ability to modulate physiological processes [16].

Pharmacokinetics and Pharmacodynamics

Absorption and distribution: MgSO_4_ undergoes absorption following administration, with two primary routes - either through the gastrointestinal tract or intravenous administration in medical settings. This dual absorption mechanism provides flexibility in therapeutic applications. Once absorbed, MgSO_4_ exhibits a wide distribution throughout the body, permeating various tissues. A notable fraction of the administered magnesium is sequestered in bones and intracellular compartments, indicating a complex distribution profile that influences its physiological effects across different anatomical regions [17].

Metabolism and elimination: The metabolic fate of magnesium primarily unfolds in the kidneys, where most of its processing occurs. Renal metabolism plays a pivotal role in determining the magnesium levels in the body. The elimination process is predominantly renal, underscoring the kidneys' significance in regulating magnesium homeostasis. Impaired renal function poses a noteworthy consideration, as compromised kidney function can impact magnesium excretion. This connection necessitates a cautious approach, particularly in patients with renal insufficiency, to prevent potential complications related to the accumulation of magnesium in the body [18].

Pharmacodynamics: The pharmacodynamics of magnesium involve its pivotal role as a cofactor in numerous enzymatic reactions. Beyond its enzymatic functions, magnesium is a crucial regulator of ion channels, exerting influence over the dynamics of calcium and potassium channels. These interactions, in turn, contribute to magnesium's effects on vascular smooth muscle tone and cardiac function. The modulation of ion channels reflects the intricate ways magnesium participates in cellular physiology, influencing cellular excitability and, consequently, broader physiological responses that extend to vascular and cardiac function. Understanding these pharmacodynamic aspects provides a foundation for comprehending magnesium's multifaceted effects in various clinical contexts [19].

Potential Benefits in Hemodynamic Stability

Vasodilatory effects: Magnesium exerts potent vasodilatory effects by interfering with calcium influx into vascular smooth muscle cells. This mechanism inhibits the normal contraction of these cells, leading to a relaxation of the blood vessels. The net result is decreased systemic vascular resistance, a critical component of blood pressure regulation. This vasodilatory action of magnesium is particularly significant in the context of laryngoscopy and tracheal intubation, as it helps prevent hypertensive responses that often accompany these procedures. Modulating vascular tone contributes to a smoother and more controlled hemodynamic response, enhancing patient safety during airway manipulation [20].

Antiarrhythmic properties: Magnesium showcases antiarrhythmic properties through its modulation of ion channels, especially those involved in cardiac electrophysiology. By influencing the flow of ions across cardiac cell membranes, magnesium helps maintain the heart's normal rhythm. This property is crucial during procedures such as laryngoscopy and tracheal intubation, where sympathetic stimulation can lead to tachycardia and arrhythmias. Magnesium's antiarrhythmic effects become particularly relevant in preventing and mitigating these irregular heartbeats, ensuring a stable cardiac rhythm during critical phases of airway management [21].

Neuroprotective effects: Magnesium's impact extends beyond cardiovascular dynamics, including neuroprotective benefits. Magnesium plays a role in safeguarding the nervous system through its influence on neuronal excitability and modulation of N-methyl-D-aspartate (NMDA) receptors. Magnesium's neuroprotective effects become essential in the perioperative period, where fluctuations in blood pressure and potential cerebrovascular incidents are concerns. This suggests a potential avenue for minimizing the risk of neurological complications during laryngoscopy and tracheal intubation, contributing to a more comprehensive approach to patient well-being [22].

Overall hemodynamic stabilization: The collective impact of MgSO_4_'s vasodilatory, antiarrhythmic, and neuroprotective effects positions it as a comprehensive strategy for maintaining hemodynamic stability. This is particularly crucial during the critical phases of laryngoscopy and tracheal intubation, where precise control of blood pressure, heart rate, and neurovascular dynamics is paramount. MgSO_4_ infusion emerges as a promising intervention addressing multiple facets of hemodynamic stability, offering a well-rounded approach to optimizing patient outcomes and safety during airway management [23].

Review of relevant studies

Methodologies Used in Previous Research

Several studies have investigated intravenous MgSO_4_ to attenuate the hemodynamic response to laryngoscopy and tracheal intubation. A randomized, double-blind comparative study found that MgSO_4_ at 30 mg/kg dose administered 90 s before laryngoscopy and intubation was superior to lignocaine in attenuating the hemodynamic response [24]. Another study concluded that prophylactic administration of MgSO_4_ was associated with a more favorable hemodynamic response than lidocaine [25]. Additionally, a double-blind, randomized controlled trial suggested that doses of MgSO^4^ less than 50 mg/kg can effectively reduce cardiovascular instability related to laryngoscopy and tracheal intubation [26,27]. These findings indicate that MgSO_4_ may effectively attenuate the hemodynamic response to laryngoscopy and tracheal intubation, with some studies suggesting it may be superior to lidocaine.

Limitations of Existing Studies

Numerous studies have delved into the effects of MgSO_4_ infusion on hemodynamic stability during laryngoscopy and tracheal intubation across diverse surgical procedures. However, certain limitations within these studies warrant consideration. A notable drawback is some studies' absence of pre-procedural assessments of difficult tracheal intubation. The oversight in evaluating the difficulty of tracheal intubation beforehand may impact the efficacy of MgSO_4_ in addressing cardiovascular instability linked to laryngoscopy and tracheal intubation [28]. Second, discrepancies in the timing of MgSO_4_ infusion were observed in certain studies, where the precise administration period was not clearly defined. This lack of clarity regarding the timing could influence the interpretation of the results [28]. Furthermore, a subset of studies did not incorporate measurements of serum magnesium levels. This omission hampers a comprehensive understanding of the pharmacokinetics and pharmacodynamics of MgSO_4_ in the specific context of laryngoscopy and tracheal intubation [28]. Another limitation pertains to the comparative aspect of some investigations. While specific studies compared MgSO_4_ to other agents, such as rocuronium, the absence of a control or placebo group was notable. This absence limits the ability to assess the effectiveness of MgSO_4_ in isolation [28]. In addition, several studies featured a restricted patient population, potentially constraining the generalizability of their findings to a broader demographic [26]. Despite these constraints, the existing body of research imparts valuable insights into the potential benefits of MgSO_4_ infusion for managing hemodynamic stability during laryngoscopy and tracheal intubation. To advance our understanding, we must acknowledge these limitations and advocate for further research characterized by more robust study designs and larger patient populations. Only through such endeavors can we confirm existing findings and unravel the nuanced role of MgSO_4_ in the specific context of laryngoscopy and tracheal intubation.

Clinical applications in ENT surgeries

Patient Selection Criteria

Cardiovascular risk factors: Identifying patients with pre-existing cardiovascular risk factors becomes crucial for considering MgSO_4_ infusion. Conditions such as hypertension, coronary artery disease, or a history of arrhythmias heighten the susceptibility to hemodynamic stress during airway manipulation. With its vasodilatory and antiarrhythmic properties, MgSO_4_ emerges as a strategic intervention to mitigate the cardiovascular challenges associated with laryngoscopy and tracheal intubation. Selecting patients with these risk factors ensures a targeted approach, addressing specific vulnerabilities and optimizing cardiovascular stability during ENT procedures [29].

Neurological considerations: Magnesium's neuroprotective effects are valuable to patient selection criteria. Individuals with a history of cerebrovascular incidents, such as strokes or transient ischemic attacks, are identified as potential candidates for magnesium infusion during ENT procedures. The neuroprotective properties of magnesium may contribute to minimizing the risk of neurological complications in this subgroup, aligning with a proactive approach to safeguarding patients with known neurological vulnerabilities during airway manipulation [30].

Renal function: Assessment of renal function assumes critical importance in the context of MgSO_4_ infusion. As magnesium is predominantly eliminated through the kidneys, patients with impaired renal function require careful consideration. Monitoring renal function ensures the appropriate excretion of magnesium and helps prevent potential complications associated with its accumulation. Individualized dosage adjustments or alternative interventions may be necessary in patients with renal insufficiency, underscoring the need for a tailored approach based on renal considerations [31].

ENT-specific factors: Tailoring the decision for MgSO_4_ infusion based on ENT-specific factors acknowledges the variability in surgical scenarios. The nature of the ENT surgery, including anticipated duration, invasiveness, and potential impact on airway dynamics, becomes integral to determining the appropriateness of magnesium infusion. Procedures with heightened hemodynamic challenges or those requiring prolonged airway management may warrant a more proactive approach to maintain stability, making MgSO_4_ a strategic choice in the overall perioperative management of ENT surgeries. This patient-centric and procedure-specific approach enhances the precision and efficacy of MgSO_4_ infusion in the ENT setting [32].

Dosage and Administration Protocols

MgSO_4_'s dosage and administration protocols (MgSO_4_) vary depending on the context and the desired effect. In the context of laryngoscopy and tracheal intubation, the typical dosage is 30 mg/kg administered intravenously over 30 s [24]. The following protocols are commonly used for treating eclampsia and prophylaxis.

Intramuscular administration: A 4 g intravenous (IV) loading dose, followed by 10 g intramuscularly (IM) and then 5 g IM every four hours [33]. IV administration of the 4 g dose was followed by a maintenance infusion of 1-2 g/hour by a controlled infusion pump [33]. For controlling seizures associated with epilepsy, glomerulonephritis, or hypothyroidism, the usual adult dose is 1 g administered IM or IV [34]. In severe cases, continuous IM administration of 1-2 g/hour may be used [34]. For managing pre-eclampsia or eclampsia, IV infusions of dilute solutions of magnesium (1%-8%) are often given in combination with other therapies [35]. Sometimes, 4-5 g (32.5-40.6 mEq) of MgSO_4_ may be administered intramuscularly into each buttock using undiluted 50% MgSO_4_ [35]. When administering MgSO_4_, it is essential to consult relevant guidelines and protocols for the specific context and desired effect.

Comparative Analysis with Other Hemodynamic Stabilizers

A comparative analysis of MgSO_4_ in conjunction with other hemodynamic stabilizers during laryngoscopy and tracheal intubation underscores the efficacy of MgSO_4_ in mitigating hemodynamic responses. Distinct findings emerge from various comparisons involving different agents. In the context of MgSO_4_ versus esmolol, one study revealed that MgSO_4_ exhibited superior hemodynamic stability and postoperative analgesia when utilized to mitigate hemodynamic extubation responses following general anesthesia [36].

Examining MgSO_4_ in comparison to dexmedetomidine and clonidine, another study found that MgSO_4_, administered at a dosage of 30 mg/kg, demonstrated significantly greater effectiveness in stabilizing hemodynamics during and after laryngoscopy and intubation compared to dexmedetomidine and clonidine [37]. A separate study focused on comparing MgSO_4_ with clonidine during cesarean section. Both drugs were associated with hemodynamic stability and favorable unawareness, but the study did not identify any superiority of one drug over the other [38]. Investigating the combination of lidocaine and MgSO_4_, a study reported that this combination preserved hemodynamic stability during general anesthesia without extending the duration of neuromuscular blockade [27].

In a randomized study comparing the effects of lidocaine and MgSO_4_ in attenuating hemodynamic responses to tracheal intubation, both drugs demonstrated good efficacy and safety for controlling hemodynamics during laryngoscopy and intubation [39]. MgSO_4_ consistently emerges as an effective hemodynamic stabilizer during laryngoscopy and tracheal intubation. While other drugs such as esmolol, dexmedetomidine, clonidine, and lidocaine also play roles in hemodynamic stability, MgSO_4_ stands out as particularly effective in specific situations, such as in the management of intracranial aneurysms [27]. These comparative studies contribute to a nuanced understanding of the diverse roles of hemodynamic stabilizers in clinical practice.

Safety and side effects of MgSO_4_ infusion

Known Adverse Effects

Hypotension: MgSO_4_'s vasodilatory effects, aimed at maintaining hemodynamic stability, carry the risk of inducing hypotension. The relaxation of vascular smooth muscle cells can lead to a decrease in blood pressure. Vigilant monitoring for hypotension is crucial, particularly in individuals with pre-existing cardiovascular conditions, as they may be more susceptible to the hypotensive effects of MgSO_4_. Proactive measures to address and manage hypotension promptly become imperative to prevent adverse outcomes during ENT surgeries [40].

Bradycardia: Excessive magnesium levels, resulting from high doses or rapid infusion, may lead to bradycardia. This poses a challenge in maintaining an optimal heart rate during ENT surgeries, where precise cardiac function is essential. Magnesium's modulation of cardiac electrophysiology, while beneficial in preventing arrhythmias, necessitates careful dosage control to avoid bradycardic episodes. Continuous heart rate monitoring becomes crucial to detect and manage bradycardia promptly, ensuring that the benefits of magnesium infusion are realized without compromising cardiac function [41].

Respiratory depression: High doses of MgSO_4_ can depress respiratory function, presenting a risk of respiratory depression. This is particularly relevant in patients with compromised lung function, where any additional respiratory compromise may have severe consequences. Close monitoring of respiratory parameters, including oxygen saturation and end-tidal carbon dioxide levels, is essential to detect signs of respiratory depression early. Adjustments in MgSO_4_ infusion rates or consideration of alternative interventions may be warranted to mitigate the risk of respiratory complications during ENT surgeries [42].

Magnesium toxicity: Accumulation of magnesium beyond therapeutic levels can lead to toxicity, presenting a spectrum of symptoms ranging from confusion and lethargy to severe manifestations such as cardiac arrest. The risk of magnesium toxicity underscores the importance of precise dosage control and vigilant monitoring of magnesium levels in the blood. Immediate intervention protocols, such as administering calcium gluconate, may be necessary to counteract magnesium toxicity and prevent life-threatening complications during ENT surgeries [43].

Renal dysfunction: While the kidneys primarily excrete magnesium, excessive levels can lead to renal dysfunction. Patients with impaired renal function, including those with pre-existing renal conditions, require close monitoring to prevent magnesium accumulation. Regular assessment of renal function through serum creatinine levels and other renal function tests becomes integral in guiding dosage adjustments and ensuring the safety of MgSO_4_ infusion in patients with compromised renal capacity. Individualized management strategies are crucial to prevent renal complications during ENT surgeries [44].

Risk-Benefit Assessment

Cardiovascular stability vs. hypotension: The delicate balance between achieving cardiovascular stability and avoiding hypotension is critical in administering MgSO_^4^_. While magnesium's vasodilatory effects contribute to cardiovascular stability, there is an inherent risk of hypotension. Striking the right balance requires careful patient selection, considering factors such as pre-existing cardiovascular conditions. Continuous blood pressure monitoring is essential during ENT surgeries to promptly identify and address hypotensive episodes. This nuanced approach ensures that the potential benefits of maintaining cardiovascular stability are optimized while minimizing the risks associated with hypotension [45].

Neuroprotective effects vs. respiratory depression: MgSO_4_'s neuroprotective effects introduce a valuable dimension to patient care but must be carefully considered alongside the risk of respiratory depression. While magnesium's influence on neuronal excitability may offer neuroprotection, higher doses can depress respiratory function. Rigorous monitoring of respiratory parameters, including oxygen saturation and end-tidal carbon dioxide levels, is imperative. Adjusting infusion rates based on real-time respiratory assessments becomes integral to mitigating the risk of respiratory depression. This dynamic interplay ensures that the neuroprotective benefits are harnessed without compromising respiratory function during ENT surgeries [46].

Renal function considerations: The impact of MgSO_4_ on renal function necessitates thorough consideration, especially in patients with pre-existing renal conditions. Assessing renal function through laboratory tests, including serum creatinine levels, informs the risk of renal dysfunction. Individualized dosing strategies, accounting for renal capacity, become essential to prevent magnesium accumulation and potential renal complications. This tailored approach ensures that the benefits of MgSO_4_ infusion are realized while minimizing the risk of adverse effects on renal function during ENT surgeries [47].

Individual patient factors: Individual patient factors, including age, comorbidities, and the specific nature of the ENT surgery, should be integral to the overall risk-benefit assessment of MgSO_4_ infusion. Age-related considerations, such as altered drug metabolism and the presence of comorbidities, can influence how an individual responds to MgSO_4_. The complexity and invasiveness of the surgical procedure further impact the risk-benefit profile. A holistic evaluation, considering these individual patient factors, ensures that MgSO_4_ infusion aligns with each patient's unique needs and characteristics, optimizing its efficacy and safety in the context of ENT surgeries [48].

Monitoring and Management Strategies

Continuous monitoring: Real-time monitoring of hemodynamic parameters is imperative during MgSO_4_ infusion. Continuous assessment of blood pressure, heart rate, and respiratory rate allows for promptly identifying deviations from the desired hemodynamic stability. This ongoing monitoring provides healthcare providers with the necessary information to make informed decisions and intervene promptly, ensuring that the patient's cardiovascular status remains within the target range throughout the infusion period [49].

Magnesium level monitoring: Regular monitoring of magnesium levels in the blood is essential to prevent excessive accumulation. This proactive approach enables healthcare providers to adjust dosage rates as needed, maintaining magnesium levels within the therapeutic range. Monitoring magnesium levels is critical in mitigating the risk of adverse effects associated with insufficient or excessive magnesium concentrations, contributing to MgSO_4_ infusion's overall safety and efficacy [50].

Respiratory monitoring: Continuous monitoring of respiratory function is crucial, encompassing parameters such as oxygen saturation and end-tidal carbon dioxide levels. This comprehensive respiratory monitoring aids in the early detection of respiratory depression, a potential adverse effect of MgSO_4_ infusion. Timely identification of changes in respiratory parameters allows for swift intervention, including adjustments to infusion rates or consideration of alternative interventions to address respiratory complications during the infusion period [51].

Renal function tests: Regular assessment of renal function through tests such as serum creatinine levels is fundamental in managing MgSO_4_ infusion. Monitoring renal function ensures the timely identification of potential renal complications, particularly in patients with pre-existing renal conditions. Individualized dosing strategies can be implemented based on renal function tests, preventing the accumulation of magnesium and minimizing the risk of adverse effects on kidney function [52].

Prompt intervention protocols: Establishing and implementing prompt intervention protocols is critical to ensuring patient safety during MgSO_4_ infusion. Protocols should address potential adverse effects, such as magnesium toxicity or hypotension. For example, having guidelines for administering calcium gluconate in the event of magnesium toxicity or protocols for adjusting infusion rates in response to hypotension ensures that healthcare providers can swiftly and effectively manage complications as they arise, contributing to a proactive and patient-centered approach to care [53].

Future directions and research recommendations

Gaps in Current Understanding

Optimal dosage and administration: The variability in dosing protocols and administration strategies for MgSO_4_ infusion underscores the necessity for well-designed studies to establish an optimal dosage and administration regimen. Tailoring these parameters to different patient populations, encompassing factors such as age, comorbidities, and the nature of ENT surgeries, is crucial. Robust research in this domain will provide a foundation for standardized protocols, ensuring the efficacy and safety of MgSO_4_ infusion across diverse clinical scenarios [54].

Patient stratification: Identifying specific patient subgroups that may derive the most significant benefit from MgSO_4_ infusion is an ongoing challenge. Stratified analyses based on factors such as age, comorbidities, and surgical complexity could offer valuable insights into the nuanced effects of MgSO_4_ in different patient populations. Understanding which patients are more likely to benefit from this intervention will contribute to personalized and targeted approaches in perioperative care, optimizing outcomes for specific groups [55].

Long-term outcomes: The limited data on the long-term effects of MgSO_4_ infusion beyond the immediate perioperative period necessitate further investigation. Research exploring its impact on postoperative recovery, the recurrence of symptoms, and overall patient outcomes will provide a comprehensive understanding of the sustained benefits or potential challenges associated with MgSO_4_ use. Long-term outcome studies are essential for guiding postoperative care strategies and informing patients about the extended implications of this intervention [56].

Comparative effectiveness: Head-to-head comparisons between MgSO_4_ and other hemodynamic stabilizers are essential for evidence-based clinical decision-making. Comparative effectiveness studies should evaluate MgSO_4_'s efficacy in maintaining hemodynamic stability and assess its safety profile compared to other commonly used interventions. This research is pivotal for clinicians when making informed choices about the most suitable hemodynamic stabilizer for specific patients and surgical contexts, contributing to the refinement of perioperative care practices [57].

Potential Areas for Further Investigation

Mechanistic studies: Conducting in-depth studies is crucial to unravel the precise mechanisms through which MgSO_4_ modulates hemodynamic stability during laryngoscopy and tracheal intubation. Understanding the intricate cellular and molecular pathways influenced by MgSO_4_ provides a foundational knowledge base for targeted interventions. Mechanistic insights can unveil specific points of intervention, guiding the development of more refined and efficient strategies for maintaining hemodynamic stability. These studies contribute not only to the optimization of MgSO_4_ use but also to a broader understanding of the physiological responses during airway manipulation [58].

Impact on surgical outcomes: Investigating the impact of MgSO_4_ infusion on surgical outcomes represents a critical avenue for research. This includes assessing its influence on intraoperative parameters, such as hemodynamic stability and anesthetic requirements, as well as postoperative outcomes, such as length of stay, complication rates, and overall recovery. A comprehensive evaluation of these factors provides a more nuanced understanding of the clinical benefits associated with MgSO_4_ use in ENT surgeries. Such studies contribute valuable evidence to guide clinical decision-making, perioperative care strategies, and patient expectations [59].

Dose-response relationships: Examining dose-response relationships is essential for refining dosage recommendations and establishing therapeutic ranges for MgSO_4_ infusion. Understanding the optimal dosage that achieves hemodynamic stability while minimizing the risk of adverse effects is crucial for safe and effective clinical use. Dose-response studies contribute to developing evidence-based guidelines, offering clinicians clear recommendations for MgSO_4_ administration tailored to specific patient populations and procedural contexts [60].

Exploration of alternative routes: Investigating alternative routes of MgSO_4_ administration, such as intramuscular or oral routes, is a promising avenue for research. Exploring these alternative routes may offer practical advantages, including ease of administration and reduced reliance on intravenous access. The feasibility and effectiveness of alternative administration routes should be evaluated, considering factors such as bioavailability and onset of action. Such studies broaden the options for MgSO_4_ use in ENT surgeries, potentially enhancing its accessibility and applicability in diverse clinical settings [61].

Implications for Clinical Practice

Guidelines and protocols: The accumulation of robust evidence supporting the use of MgSO_4_ in ENT surgeries should drive the development of clinical guidelines and protocols. These guidelines can serve as a valuable resource for healthcare providers, offering standardized and evidence-based recommendations for administering MgSO_4_. Clear protocols can enhance consistency in practice, ensuring that MgSO_4_ is used optimally and safely across different clinical settings. These guidelines provide a framework for decision-making and contribute to the overall quality and standardization of care in ENT surgeries [62].

Educational initiatives: Integrating research findings into educational initiatives is crucial for disseminating knowledge about MgSO_4_ infusion's potential benefits and risks. Educational programs targeted at healthcare professionals, including anesthesiologists, surgeons, and nursing staff, facilitate awareness and understanding of the role of MgSO_4_ in maintaining hemodynamic stability during airway manipulation. Continuous education ensures that healthcare providers are well-informed about the latest evidence, fostering a culture of evidence-based practice and supporting the integration of MgSO_4_ into routine perioperative care [63].

Informed consent practices: Enhancing informed consent practices is essential as MgSO_4_ becomes a more established option in ENT surgeries. Educating patients about MgSO_4_'s rationale, potential benefits, and known risks empowers them to participate in shared decision-making. Informed consent discussions should include information about the role of MgSO_4_ in maintaining hemodynamic stability and any relevant considerations specific to the patient's medical history. Transparent communication ensures that patients are well-informed about the potential implications of MgSO_4_ infusion, promoting patient autonomy and satisfaction [64].

Multidisciplinary collaboration: Future research and implementation efforts should prioritize and encourage multidisciplinary collaboration between anesthesiologists, surgeons, and researchers. A holistic approach to patient care involves leveraging the expertise of different healthcare professionals to optimize outcomes in ENT surgeries. Collaboration facilitates the exchange of knowledge, ensures comprehensive patient management, and allows for the integration of MgSO_4_ into perioperative protocols. Multidisciplinary teamwork promotes a patient-centered approach, where each healthcare professional contributes unique insights to enhance the overall quality of care in ENT surgeries [65].

## Conclusions

In conclusion, the comprehensive review of MgSO_4_ infusion's impact on hemodynamic stability during laryngoscopy and tracheal intubation in ENT surgeries reveals a multifaceted pharmacological profile with promising potential. Exploring its vasodilatory, antiarrhythmic, and neuroprotective effects suggests a valuable role in mitigating the challenges posed by airway manipulation. However, integrating MgSO_4_ into clinical practice demands a nuanced approach, considering patient selection criteria, optimal dosage protocols, and a careful risk-benefit assessment to navigate its associated adverse effects. The implications for ENT surgery are substantial, offering the prospect of enhanced hemodynamic management and individualized approaches tailored to patient-specific factors. While recognizing the advancements in perioperative care that MgSO_4_ could bring, it is essential to emphasize the ongoing nature of research in this domain. As clinicians contemplate the incorporation of MgSO_4_ into their practice, a vigilant and evidence-based approach guided by emerging clinical guidelines will contribute to its safe and effective utilization. This review encourages continued collaboration among researchers, anesthesiologists, and surgeons to refine the understanding of MgSO_4_'s role, ensuring its optimal application and, ultimately, the improved care of ENT surgery patients.
